# Supplementary material for: Factorial structure, reliability, and construct validity of the Generalized Anxiety Disorder 7-item (GAD-7): Evidence from Malaysia
Source: PLoS One. 2023 May 11;18(5):e0285435. doi: 10.1371/journal.pone.0285435 (PMC10174505; doi:10.1371/journal.pone.0285435)
Supplement: S1 Table — (DOCX) [file pone.0285435.s001.docx]

| Table S1 | | | |
| --- | --- | --- | --- |
| Summary the factor structure of the Generalized Anxiety Disorder 7-item (GAD-7) | | | |
| No. | Author (Year) | Sample | Model |
| 1 | Monteiro et al. (2022) | 746 Brazilians (18–72 years) | One-factor model with 7 items |
| 2 | Pranckeviciene et al. (2022) | 1,368 Lithuanian university students |  |
| 3 | Soto-Balbuena et al. (2021) | 385 Spanish pregnant women |  |
| 4 | Byrd-Bredbenner et al. (2020) | 4,128 American university students |  |
| 5 | Nyongesa et al. (2020) | 450 Kenyan adults living with HIV |  |
| 6 | Tiirikainen et al. (2019) | 111,171 Finnish adolescents |  |
| 7 | Omani-Samani et al. (2018) | 539 Iranians with infertility problems |  |
| 8 | Hinz et al. (2017) | 9,721 Germans (18–80 years) |  |
| 9 | Sawaya et al. (2016) | 186 Lebanese adult psychiatric outpatients |  |
| 10 | Sousa et al. (2015) | 100 patients with generalized anxiety disorder |  |
| 11 | Sun et al. (2021) | 67,821 adolescents aged 10–17 years |  |
| 12 | Zhong et al. (2015) | 2,978 Peruvian pregnant women |  |
| 13 | Zhang et al. (2021) | 1,021 Chinese medical university students | Modified one-factor model with 7 items:  error covariance between items 3 and 7 |
| 14 | Woon et al. (2020) | 300 diabetic outpatients | Modified one-factor model:  error covariance between items 1 and 2, and items 2 and 3 |
| 15 | Bártolo et al. (2017) | 1,031 Portuguese university students | Modified one-factor model:  error covariance between items 4 and 5, and items 5 and 6 |
| 16 | Ryan et al. (2013) | 23,672 British patients |  |
| 17 | Johnson et al. (2019) | 1,201 Norwegian heterogeneous psychiatric patients | Modified one-factor model with 7 items:  error covariance between items 4 and 5, items 5 and 6, and items 4 and 6 |
| 18 | Lee and Kim (2019) | 582 Korean university students |  |
| 19 | Parkerson et al. (2015) | 1,057 American and Canadian university students |  |
| 20 | Kertz et al. (2013) | 232 American acute psychiatric patients |  |
| 21 | Dhira et al. (2021) | 677 Bangladeshi university students | Modified one-factor model with 7 items:  error covariance between items 1 and 2, items 4 and 5, and items 5 and 6 |
| 22 | Ong et al. (2021) | 4,323 American patients | Two-factor model with 7 items:  items 1, 2, 3, and 7 loaded on cognitive factor, and items 4 to 6 loaded on somatic factor |
| 23 | Moreno et al. (2019) | 1,255 Spanish patients with emotional disorders |  |
| 24 | Beard and Björgvinsson (2014) | 1,082 American heterogeneous psychiatric patients | Two-factor model with 7 items:  items 1, 2, 3, and 7 loaded on cognitive and emotional experience of anxiety factor, and items 4 to 6 loaded on physical experience of restlessness and irritability factor |
| 25 | Doi et al. (2018) | 1,447 Japanese adults with and without self-reported psychiatric diagnostic status | Second-order factor model with 7 items:  two first-order factors (items 1, 2, 3, and 7 loaded on cognitive and emotional experience of anxiety factor, and items 4 to 6 loaded on physical experience of restlessness factor) and a single second-order factor |

References

Bártolo, A., Monteiro, S., & Pereira, A. (2017). Factor structure and construct validity of the Generalized Anxiety Disorder 7-item (GAD-7) among Portuguese college students. *Cadernos de Saúde Pública, 33*(9), Article e00212716. <https://doi.org/10.1590/0102-311x00212716>

Beard, C., & Björgvinsson, T. (2014). Beyond generalized anxiety disorder: Psychometric properties of the GAD-7 in a heterogeneous psychiatric sample. *Journal of Anxiety Disorders, 28*(6), 547–552. <https://doi.org/10.1016/j.janxdis.2014.06.002>

Byrd-Bredbenner, C., Eck, K., & Quick, V. (2020). Psychometric properties of the Generalized Anxiety Disorder-7 and Generalized Anxiety Disorder-Mini in United States university students. *Frontiers in Psychology,* *11*, Article 550533. <https://doi.org/10.3389/fpsyg.2020.550533>

Dhira, T. A., Rahman, M. A., Sarker, A. R., & Mehareen, J. (2021). Validity and reliability of the Generalized Anxiety Disorder-7 (GAD-7) among university students of Bangladesh. *PLoS ONE, 16(*12), Article e0261590. <https://doi.org/10.1371/journal.pone.0261590>

Doi, S., Ito, M., Takebayashi, Y., Muramatsu, K., & Horikoshi, M. (2018). Factorial validity and invariance of the 7-item Generalized Anxiety Disorder Scale (GAD-7) among populations with and without self-reported psychiatric diagnostic status. *Frontiers in Psychology, 9*, Article 1741. <https://doi.org/10.3389/fpsyg.2018.01741>

Hinz, A., Klein, A. M., Brähler, E., Glaesmer, H., Luck, T., Riedel-Heller, S. G., Wirkner, K., & Hilbert, A. (2017). Psychometric evaluation of the Generalized Anxiety Disorder Screener GAD-7, based on a large German general population sample. *Journal of Affective Disorders, 210*, 338–344. <https://doi.org/10.1016/j.jad.2016.12.012>

Johnson, S. U., Ulvenes, P. G., Øktedalen, T., & Hoffart, A. (2019). Psychometric properties of the General Anxiety Disorder 7-Item (GAD-7) Scale in a heterogeneous psychiatric sample. *Frontiers in Psychology, 10*, Article 1713. <https://doi.org/10.3389/fpsyg.2019.01713>

Kertz, S., Bigda-Peyton, J., & Bjorgvinsson, T. (2013). Validity of the Generalized Anxiety Disorder-7 scale in an acute psychiatric sample. *Clinical psychology & psychotherapy, 20*(5), 456–464. <https://doi.org/10.1002/cpp.1802>

Lee, B., & Kim, Y. E. (2019). The psychometric properties of the Generalized Anxiety Disorder scale (GAD-7) among Korean university students. P*sychiatry and Clinical Psychopharmacology, 29*(4), 864–871. <https://doi.org/10.1080/24750573.2019.1691320>

Monteiro, R. P., Nascimento, B. S., Monteiro, T. M. C., da Silva, P. D. G., & Ferreira, A. J. C. (2022). Psychometric evidence of the 7-Item Generalized Anxiety Disorder Questionnaire in Brazil. *International Journal of Mental Health and Addiction, 20*(2), 1023–1034. <https://doi.org/10.1007/s11469-020-00423-9>

Moreno, E., Muñoz-Navarro, R., Medrano, L. A., González-Blanch, C., Ruiz-Rodríguez, P., Limonero, J. T., Moretti, K. S., Cano-Vindel, A., & Moriana, J. A. (2019). Factorial invariance of a computerized version of the GAD-7 across various demographic groups and over time in primary care patients. *Journal of Affective Disorders, 252*, 114–121. <https://doi.org/10.1016/j.jad.2019.04.032>

Nyongesa, M. K., Mwangi, P., Koot, H. M., Cuijpers, P., Newton, C. R. J. C., & Abubakar, A. (2020). The reliability, validity and factorial structure of the Swahili version of the 7-item generalized anxiety disorder scale (GAD-7) among adults living with HIV from Kilifi, Kenya. *Annals of General Psychiatry, 19*, Article 62. <https://doi.org/10.1186/s12991-020-00312-4>

Omani-Samani, R., Maroufizadeh, S., Ghaheri, A., & Navid, B. (2018). Generalized Anxiety Disorder-7 (GAD-7) in people with infertility: A reliability and validity study. Middle East Fertility Society Journal*, 23*(4), 446–449. <https://doi.org/10.1016/j.mefs.2018.01.013>

Ong, C. W., Pierce, B. G., Klein, K. P., Hudson, C. C., Beard, C., & Björgvinsson, T. (2021). Longitudinal measurement invariance of the PHQ-9 and GAD-7. *Assessment*. Advance online publication. <https://doi.org/10.1177/10731911211035833>

Parkerson, H. A., Thibodeau, M. A., Brandt, C. P., Zvolensky, M. J., & Asmundson, G. J. G. (2015). Cultural-based biases of the GAD-7. *Journal of Anxiety Disorders, 31*, 38–42. <https://doi.org/10.1016/j.janxdis.2015.01.005>

Pranckeviciene, A., Saudargiene, A., Gecaite-Stonciene, J., Liaugaudaite, V., Griskova-Bulanova, I., Simkute, D., Naginiene, R., Dainauskas, L. L., Ceidaite, G., & Burkauskas, J. (2022). Validation of the Patient Health Questionnaire-9 and the Generalized Anxiety Disorder-7 in Lithuanian student sample. *PLoS ONE, 17*(1), Article e0263027. <https://doi.org/10.1371/journal.pone.0263027>

Ryan, T. A., Bailey, A., Fearon, P., & King, J. (2013). Factorial invariance of the Patient Health Questionnaire and Generalized Anxiety Disorder Questionnaire. *British Journal of Clinical Psychology, 52*(4), 438–449. <https://doi.org/10.1111/bjc.12028>

Sawaya, H., Atoui, M., Hamadeh, A., Zeinoun, P., & Nahas, Z. (2016). Adaptation and initial validation of the Patient Health Questionnaire – 9 (PHQ-9) and the Generalized Anxiety Disorder – 7 Questionnaire (GAD-7) in an Arabic speaking Lebanese psychiatric outpatient sample. *Psychiatry Research, 239*, 245–252. <https://doi.org/10.1016/j.psychres.2016.03.030>

Soto-Balbuena, C., Rodríguez-Muñoz, M. F., & Le, H.-N. (2021). Validation of the Generalized Anxiety Disorder Screener (GAD-7) in Spanish pregnant women. *Psicothema, 33*(1), 164–170. <https://doi.org/10.7334/psicothema2020.167>

Sousa, T. V., Viveiros, V., Chai, M. V., Vicente, F. L., Jesus, G., Carnot, M. J., Gordo, A. C., & Ferreira, P. L. (2015). Reliability and validity of the Portuguese version of the Generalized Anxiety Disorder (GAD-7) scale. *Health and Quality of Life Outcomes, 13*, Article 50. <https://doi.org/10.1186/s12955-015-0244-2>

Tiirikainen, K., Haravuori, H., Ranta, K., Kaltiala-Heino, R., & Marttunen, M. (2019). Psychometric properties of the 7-item Generalized Anxiety Disorder Scale (GAD-7) in a large representative sample of Finnish adolescents. *Psychiatry Research, 272*, 30–35. <https://doi.org/10.1016/j.psychres.2018.12.004>

Woon, L. S., Hatta, S., & Norlaila, M. (2020). Factor Structure of The Malay-Version Generalized Anxiety Disorder-7 (GAD-7) questionnaire among patients with diabetes mellitus. *Medicine & Health*, *15*, 208-217. <https://doi.org/10.17576/MH.2020.1501.19>

Zhang, C., Wang, T., Zeng, P., Zhao, M., Zhang, G., Zhai, S., Meng, L., Wang, Y., & Liu, D. (2021). Reliability, validity, and measurement invariance of the General Anxiety Disorder Scale among Chinese medical university students. *Frontiers in Psychiatry, 12*, Article 648755. <https://doi.org/10.3389/fpsyt.2021.648755>

Zhong, Q.-Y., Gelaye, B., Zaslavsky, A. M., Fann, J. R., Rondon, M. B., Sánchez, S. E., & Williams, M. A. (2015). Diagnostic validity of the Generalized Anxiety Disorder - 7 (GAD-7) among pregnant women. *PLoS ONE, 10*(4), Article e0125096. <https://doi.org/10.1371/journal.pone.0125096>
